# Development and validation of an intraoperative hypothermia nomograph model for patients undergoing video-assisted thoracoscopic lobectomy: a retrospective study

**DOI:** 10.1038/s41598-024-66222-7

**Published:** 2024-07-02

**Authors:** Fuhai Xia, Qiang Li, Liqin Xu, Xi Chen, Gen Li, Li Li, Zhineng Cheng, Jie Zhang, Chaoliang Deng, Jing Li, Rui Chen

**Affiliations:** Operating Room, The Central Hospital of Wuhan, Tongji Medical College, Huazhong University of Science and Technology, Wuhan, 430014, China

**Keywords:** Video-assisted thoracoscopic lobectomy, Intraoperative hypothermia, Risk factors, Prediction model, Nomogram, Risk factors, Lung cancer

## Abstract

This study aimed to develop and internally validate a nomogram model for assessing the risk of intraoperative hypothermia in patients undergoing video-assisted thoracoscopic (VATS) lobectomy. This study is a retrospective study. A total of 530 patients who undergoing VATS lobectomy from January 2022 to December 2023 in a tertiary hospital in Wuhan were selected. Patients were divided into hypothermia group (n = 346) and non-hypothermia group (n = 184) according to whether hypothermia occurred during the operation. Lasso regression was used to screen the independent variables. Logistic regression was used to analyze the risk factors of hypothermia during operation, and a nomogram model was established. Bootstrap method was used to internally verify the nomogram model. Receiver operating characteristic (ROC) curve was used to evaluate the discrimination of the model. Calibration curve and Hosmer Lemeshow test were used to evaluate the accuracy of the model. Decision curve analysis (DCA) was used to evaluate the clinical utility of the model. Intraoperative hypothermia occurred in 346 of 530 patients undergoing VATS lobectomy (65.28%). Logistic regression analysis showed that age, serum total bilirubin, inhaled desflurane, anesthesia duration, intraoperative infusion volume, intraoperative blood loss and body mass index were risk factors for intraoperative hypothermia in patients undergoing VATS lobectomy (*P* < 0.05). The area under ROC curve was 0.757, 95% CI (0.714–0.799). The optimal cutoff value was 0.635, the sensitivity was 0.717, and the specificity was 0.658. These results suggested that the model was well discriminated. Calibration curve has shown that the actual values are generally in agreement with the predicted values. Hosmer–Lemeshow test showed that χ2 = 5.588, *P* = 0.693, indicating that the model has a good accuracy. The DCA results confirmed that the model had high clinical utility. The nomogram model constructed in this study showed good discrimination, accuracy and clinical utility in predicting patients with intraoperative hypothermia, which can provide reference for medical staff to screen high-risk of intraoperative hypothermia in patients undergoing VATS lobectomy.

## Introduction

With the increasing promotion of population aging and urbanization, lung cancer has become the world’s largest malignant tumor and a serious threat to human health^1^. According to Global Cancer Statistics 2020, the global new incidence of lung cancer is 11.4%, ranking second in new cancer incidence and first in cancer mortality^2^. Lung cancer ranks first in the incidence and mortality of cancer in China^1^. In recent years, with the improvement of people’s living standards and health care awareness, the detection rate of early lung cancer is also increasing year by year. The preferred treatment is video-assisted thoracoscopic (VATS) lobectomy^3^. The use of VATS lobectomy was reported to have increased significantly, from 8.0% in 2003 to 54.7% in 2014^4^. Compared with traditional thoracotomy, VATS has the characteristics of less trauma, shorter duration of chest tube, less postoperative pain, and shorter hospital stay^5^. In addition, VATS can also reduce patients’ medical costs^6^, perioperative morbidity and mortality^7^, significantly improve patients’ postoperative quality of life and promote rapid recovery. However, due to the long operation and anesthesia time, patients are prone to hypothermia during VATS surgery^8^.

Intraoperative hypothermia is defined as the core body temperature of the patient below 36°C caused by any reason during surgery, which is a common surgical complication^9,10^. It has been reported that the overall incidence of intraoperative hypothermia in some parts of China is 44.3%^11^. The incidence of intraoperative hypothermia was 78.3% in patients undergoing thoracic surgery^12^ and 50–72.7% in patients undergoing VATS lung tumor resection^13^. Intraoperative hypothermia is associated with a number of adverse outcomes, including postoperative cardiovascular events^14^, perioperative bleeding, drug metabolism disorders, wound infection, increased risk of postoperative delirium^15,16^, and increased risk of deep vein thrombosis^17^. It may also lead to prolonged recovery time from anesthesia^18^, reduced thermal comfort and satisfaction of patients, and increased medical costs^6^. Therefore, maintaining a stable intraoperative body temperature is an important measure to reduce intraoperative, postoperative and anesthesia complications.

Previous studies have shown that risk factors for intraoperative hypothermia in patients undergoing VATS surgery include the following^3,19^. Demographic characteristics: including age, gender, body mass index (BMI). Physiological factors: including underlying diseases, preoperative body temperature, preoperative blood pressure, hemoglobin levels, white blood cells, platelets, etc. Operative related factors: including operation duration, operating room temperature, intraoperative infusion, surgical position, etc.

Therefore, early and effective prevention of hypothermia during the operation is the key to better temperature management. Since there are many risk factors for intraoperative hypothermia in patients undergoing VATS lobectomy, accurate prediction and early intervention are particularly important in the prevention of intraoperative hypothermia. However, current studies on intraoperative hypothermia mainly focus on patients undergoing abdominal surgery^20^, patients undergoing lung cancer resection^21^, and orthopedic surgery^22^. There are no reports on the development and validation of intraoperative hypothermia prediction model for patients undergoing VATS lobectomy. In recent years, in the field of hypothermia pre-control research, prediction models of intraoperative hypothermia have developed rapidly, but the predictive ability of most models remains to be explored^23^. This study aimed to develop and internally validate a nomogram model for assessing the risk of intraoperative hypothermia in patients undergoing VATS lobectomy, so as to provide reference for medical staff to screen high-risk of intraoperative hypothermia in patients undergoing VATS lobectomy.

## Methods

### Study design

**Figure 1:**
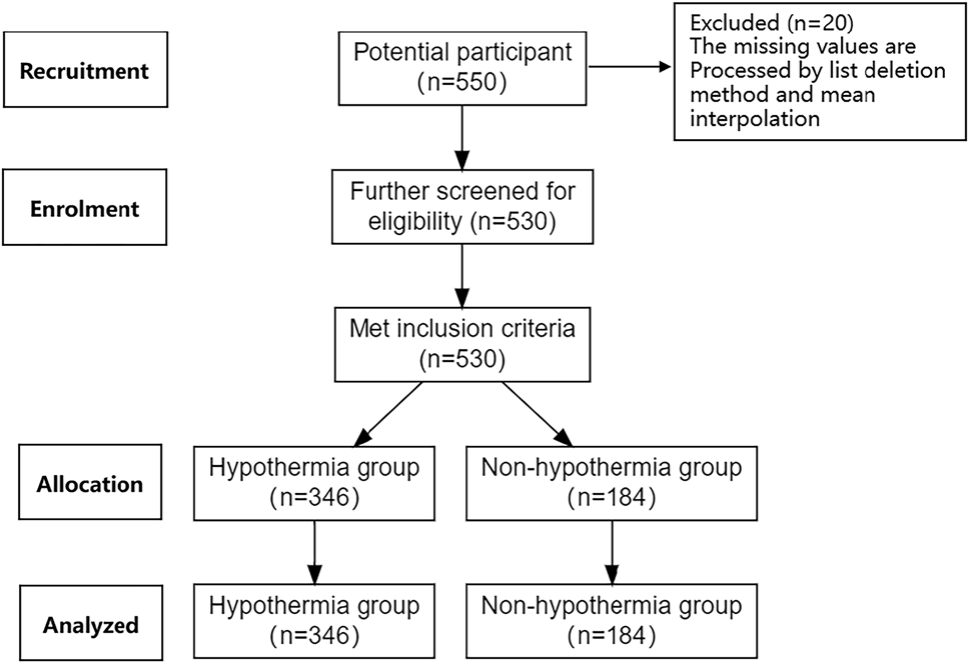
Flowchart of the study.

This study is a retrospective study and follows TRIPOD statement. A total of 530 patients who undergoing VATS lobectomy from January 2022 to December 2023 in a tertiary hospital in Wuhan were selected. Inclusion criteria: (1) had indications for VATS lobectomy^24^; (2) age ≥ 18 years; (3) elective surgery; (4) communicate fluently; (5) informed consent and voluntary participation in this study. (6) body anaesthesia and (7) complete medical records. Exclusion criteria: (1) impaired thermoregulation; (2) the nasal cavity has undergone surgery or has abnormalities that make it impossible to monitor nasopharyngeal temperature; (3) intraoperative temporary change to open surgery; (4) massive bleeding, shock, respiratory and cardiac arrest occurred during the operation and (5) planned hypothermia surgery. Figure 1 shows the flowchart of the study.

### Sample

Based on literature review and expert experience, a total of 27 risk factors were included in this study. It has been reported that the sample size should be 10 to 15 times that of the independent variable (risk factors) in order to obtain reliable parameter estimation^25^. Given a 10–20% sample loss, the sample size should be 297–482, which means 530 samples match the criteria.

### Ethical consideration

This study was approved by the Wuhan Central Hospital ethics committee (number WHZXKYL2024-011) and followed the principles of Declaration of Helsinki. This study was approved by the Ethics Committee of Wuhan Central Hospital and exempted from informed consent.

### Measures

This study designed a data collection table, including the following four parts. Demographic characteristics: including gender, age, BMI, preoperative body temperature, smoking history, drinking history, combined hypertension, combined diabetes, transfusion history and operation history. Intraoperative data: including surgical spot, intraoperative infusion volume and intraoperative blood loss. Anesthesia data: including American society of anesthesiologists (ASA), inhaled desflurane and anesthesia duration. Biochemical indexes: including serum total bilirubin (TBIL), albumin, hemoglobin, platelet, red blood cells, white blood cells, D-Dimer, creatinine, urea, prothrombin time and activated partial thromboplastin time.

### Data collection

All data were collected retrospectively from the hospital information system. All biochemical indexes were completed within 3 days before operation. Intraoperative hypothermia is determined by real-time monitoring of the patient’s body temperature. Intraoperative hypothermia was determined when the patient’s temperature at any time was less than 36 °C^26^. The nasopharynx was selected as the part to measure body temperature, and the temperature data of patients were obtained through the monitor in this study^26^. The monitor automatically measures and uploads the patient’s nasopharyngeal temperature every 5 min. All patients were kept warm with quilts. The study data were entered and checked by two people. The missing values are processed by list deletion method and mean interpolation. We excluded 20 cases by list deletion method and eventually included 530 cases.

### Data analysis

All data analysis was performed using SPSS version 26.0 and R version 4.3.2. Categorical variables were described by frequency and percentage, and comparison among groups was used by chi-square test (χ^2^). Continuous variables were described by mean and standard deviation (SD), and comparison among groups was used by T-test. Kolmogorov–Smirnov test was used for normality test. Continuous variables of skewed distribution were described by M (P25, P75), and comparison among groups was used by Mann–Whitney U test. Lasso regression was used to screen the independent variables. Logistic regression was used to construct the prediction model. R version 4.3.2 was used to construct the nomogram and visualize the prediction model. Bootstrap method was used to repeat 1000 times for internal validation of the nomogram model. Receiver operating characteristic (ROC) curve was used to evaluate the discrimination of the model. Calibration curve and Hosmer Lemeshow test were used to evaluate the accuracy of the model. Decision curve analysis (DCA) was used to evaluate the clinical utility of the model. All statistical tests were conducted by two-sided tests, and *P* values of < 0.05 indicated statistical significance.

## Results

### Sample characteristics

A total of 530 patients who underwent VATS lobectomy were included in this study. Of the 530 participants, 290 were male, accounting for 54.72%. The median age was 63.00 years. The superior lobe of the left lung was surgically removed in 142 cases (26.79%), the inferior lobe of the left lung in 105 cases (19.81%), the superior lobe of the right lung in 164 cases (30.94%), the middle lobe of the right lung in 22 cases (4.15%), and the inferior lobe of the right lung in 97 cases. Intraoperative hypothermia occurred in 346 of 530 patients undergoing VATS lobectomy (65.28%). More sample characteristics are shown in Table 1.

**Table 1:** Baseline characteristics of the patients.

Variable	Total (n = 530)	Non-hypothermia group (n = 184)	Hypothermia group (n = 346)	Statistic	*P* value
Hemoglobin [g/L, (Mean ± SD)]	133.16 ± 15.75	134.27 ± 14.76	132.57 ± 16.24	t = 1.181	0.238
TBIL [μmol/L, Mean ± SD]	13.54 ± 6.13	14.40 ± 6.76	13.09 ± 5.72	t = 2.348	0.019
Age [Year, M (Q₁, Q₃)]	63.00 (54.00–68.00)	60.00 (46.75–68.00)	64.00 (56.25–69.00)	Z = − 3.877	< 0.001
Preoperative body temperature [°C, M (Q₁, Q₃)]	36.50 (36.40–36.60)	36.50 (36.40–36.52)	36.50 (36.40–36.60)	Z = − 1.688	0.091
Red blood cells [10^12^/L, M (Q₁, Q₃)]	4.30 (4.03–4.66)	4.33 (4.08–4.70)	4.30 (4.00–4.63)	Z = − 1.680	0.093
White blood cells [10^9^/L, M (Q₁, Q₃)]	5.73 (4.67–6.90)	5.72 (4.83–6.95)	5.73 (4.58–6.89)	Z = − 0.666	0.506
Platelet [10^9^/L, M (Q₁, Q₃)]	209.00 (178.00–251.00)	204.50 (177.00–253.50)	209.50 (178.25–250.75)	Z = − 0.290	0.772
D-Dimer [μg/ml, M (Q₁, Q₃)]	0.36 (0.19–0.51)	0.38 (0.19–0.51)	0.36 (0.20–0.51)	Z = − 0.061	0.952
Creatinine [μmol/L, M (Q₁, Q₃)]	68.20 (56.00–76.00)	66.50 (55.15–75.50)	68.37 (56.12–77.70)	Z = − 0.992	0.321
Urea [μmol/L, M (Q₁, Q₃)]	340.00 (272.50–399.00)	345.19 (275.00–397.00)	335.00 (272.50–399.00)	Z = − 0.781	0.435
Prothrombin time [s, M (Q₁, Q₃)]	11.20 (10.60–12.10)	11.30 (10.60–12.10)	11.10 (10.50–12.00)	Z = − 0.659	0.510
Albumin [g/L, M (Q₁, Q₃)]	42.53 (39.70–45.20)	42.60 (40.45–46.00)	42.53 (39.32–44.88)	Z = − 2.379	0.017
Activated partial thromboplastin time [s, M (Q₁, Q₃)]	26.90 (24.00–29.80)	26.85 (24.30–30.30)	26.90 (23.60–29.70)	Z = − 0.833	0.405
Gender [n (%)]				χ^2^ = 0.181	0.671
Male	290 (54.72)	103 (55.98)	187 (54.05)		
Female	240 (45.28)	81 (44.02)	159 (45.95)		
BMI [n (%)]				χ^2^ = 8.188	0.017
18.5–23.9 kg/m^2^	275 (51.89)	82 (44.57)	193 (55.78)		
< 18.5 kg/m^2^	56 (10.57)	27 (14.67)	29 (8.38)		
> 23.9 kg/m^2^	199 (37.55)	75 (40.76)	124 (35.84)		
Smoking history [n (%)]				χ^2^ = 1.349	0.245
No	384 (72.45)	139 (75.54)	245 (70.81)		
Yes	146 (27.55)	45 (24.46)	101 (29.19)		
Drinking history [n (%)]				χ^2^ = 0.272	0.602
No	469 (88.49)	161 (87.50)	308 (89.02)		
Yes	61 (11.51)	23 (12.50)	38 (10.98)		
Combined hypertension [n (%)]				χ^2^ = 4.364	0.037
No	331 (62.45)	126 (68.48)	205 (59.25)		
Yes	199 (37.55)	58 (31.52)	141 (40.75)		
Combined diabetes [n (%)]				χ^2^ = 0.771	0.380
No	451 (85.09)	160 (86.96)	291 (84.10)		
Yes	79 (14.91)	24 (13.04)	55 (15.90)		
Transfusion history [n (%)]				χ^2^ = 0.000	1.000
No	521 (98.3)	181 (98.37)	340 (98.27)		
Yes	9 (1.7)	3 (1.63)	6 (1.73)		
Operation history [n (%)]				χ^2^ = 4.320	0.038
No	261 (49.25)	102 (55.43)	159 (45.95)		
Yes	269 (50.75)	82 (44.57)	187 (54.05)		
ASA [n (%)]				χ^2^ = 4.285	0.117
I	17 (3.21)	9 (4.89)	8 (2.31)		
II	359 (67.74)	116 (63.04)	243 (70.23)		
III	154 (29.06)	59 (32.07)	95 (27.46)		
Surgical spot [n (%)]				χ^2^ = 8.487	0.075
Superior lobe of left lung	142 (26.79)	57 (30.98)	85 (24.57)		
Inferior lobe of left lung	105 (19.81)	39 (21.20)	66 (19.08)		
Superior lobe of right lung	164 (30.94)	58 (31.52)	106 (30.64)		
Middle lobe of right lung	22 (4.15)	3 (1.63)	19 (5.49)		
Inferior lobe of right lung	97 (18.3)	27 (14.67)	70 (20.23)		
Inhaled desflurane [n (%)]				χ^2^ = 17.286	< 0.001
No	164 (30.94)	78 (42.39)	86 (24.86)		
Yes	366 (69.06)	106 (57.61)	260 (75.14)		
Anesthesia duration [n (%)]				χ^2^ = 36.614	< 0.001
≤ 180 min	103 (19.43)	62 (33.70)	41 (11.85)		
> 180 min	427 (80.57)	122 (66.30)	305 (88.15)		
Intraoperative infusion volume [n (%)]				χ^2^ = 38.058	< 0.001
≤ 1500 ml	289 (54.53)	134 (72.83)	155 (44.80)		
> 1500 ml	241 (45.47)	50 (27.17)	191 (55.20)		
Intraoperative blood loss [n (%)]				χ^2^ = 18.364	< 0.001
≤ 100 ml	393 (74.15)	157 (85.33)	236 (68.21)		
> 100 ml	137 (25.85)	27 (14.67)	110 (31.79)		

### Univariate analysis of intraoperative hypothermia

Patients were divided into hypothermia group (n = 346) and non-hypothermia group (n = 184) according to whether hypothermia occurred during the operation. The results showed that TBIL, age, albumin, BMI, combined hypertension, operation history, inhaled desflurane, anesthesia duration, intraoperative infusion volume and intraoperative blood loss, differences were statistically significant (*P* < 0.05). However, there was no significant difference in red blood cells, ASA and surgical spot (*P* > 0.05). More sample characteristics are shown in Table 1.

### Lasso and logistic regression analysis

**Figure 2:**
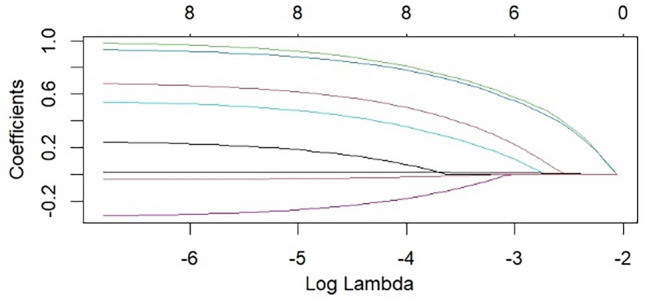
LASSO coefficient profiles of intraoperative hypothermia.

**Figure 3:**
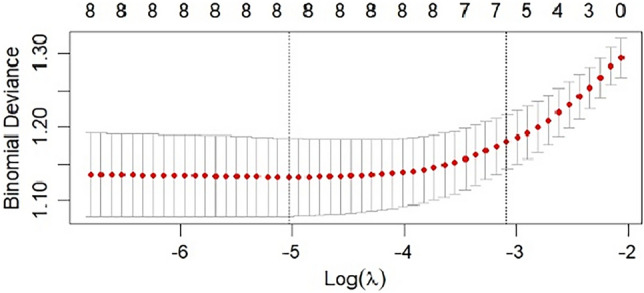
Tenfold cross-cross validation fitting profiles.

Ten variables with statistically significant in the univariate analysis were included in Lasso regression for variable screening. tenfold cross-validation was used to select the optimal lambda parameter values. The lambda parameter value with the smallest cross-validation error was taken as the optimal value of the model, and the number of variables at this time was calculated (See Figs. 2,3). Lasso regression has shown that Seven variables including TBIL, age, BMI, inhaled desflurane, anesthesia duration, intraoperative infusion volume and intraoperative blood loss were screened. The curves in Fig. 2 represent the change trajectory of independent variable coefficients. The lower x-axis represents Log Lambda value, the upper x-axis represents the number of nonzero coefficients, and the y-axis represents the penalty coefficient. Figure 3 was a tenfold cross-validation fit curve. The lower x-axis represents Log Lambda value, the upper x-axis represents the number of nonzero coefficients, and the y-axis represents binomial deviance.

Further, the intraoperative hypothermia was taken as the dependent variable (non-hypothermia = 0, hypothermia = 1), and 7 variables selected by Lasso regression were incorporated into Logistic regression for analysis. Table 2 showed how the independent variables were assigned. Logistic regression analysis showed that age, TBIL, inhaled desflurane, anesthesia duration, intraoperative infusion volume, intraoperative blood loss and body mass index were risk factors for intraoperative hypothermia in patients undergoing VATS lobectomy (*P* < 0.05). More sample characteristics are shown in Table 3.

**Table 2:** Independent variables assignments.

Variables	Independent variables assignments
Age	Raw data entry
TBIL	Raw data entry
Inhaled desflurane	0 = “no”; 1 = “yes”
Anesthesia duration	0 = “ ≤ 180 min”; 1 = “ > 180 min”
Intraoperative infusion volume	0 = “ ≤ 1500 ml”; 1 = “ > 1500 ml”
Intraoperative blood loss	0 = “ ≤ 100 ml”; 1 = “ > 100 ml”
BMI	0 = “18.5 ~ 23.9 kg/m2”; 1 = “BMI < 18.5 kg/m2”; 2 = “BMI > 23.9 kg/m2”

**Table 3:** Logistic regression analysis of intraoperative hypothermia in patients undergoing VAST lobectomy.

Variables	*β*	Standard error	Waldχ2	*P*	*OR*	95% *CI*
Age	0.020	0.008	7.001	0.008	1.020	1.005–1.035
TBIL	− 0.041	0.017	5.987	0.014	0.959	0.928–0.992
Inhaled desflurane	0.708	0.215	10.818	0.001	2.029	1.331–3.093
Anesthesia duration	0.974	0.257	14.307	0.000	2.648	1.599–4.385
Intraoperative infusion volume	0.962	0.214	20.220	0.000	2.617	1.721–3.981
Intraoperative blood loss	0.530	0.265	4.012	0.045	1.699	1.011–2.855
BMI < 18.5 kg/m^2^	− 0.369	0.352	1.095	0.295	0.692	0.347–1.380
BMI > 23.9 kg/m^2^	-0.622	0.220	7.980	0.005	0.537	0.349–0.827
Constant	-1.444	0.542	7.102	0.008	0.236	–

### Development of intraoperative hypothermia nomogram model

**Figure 4:**
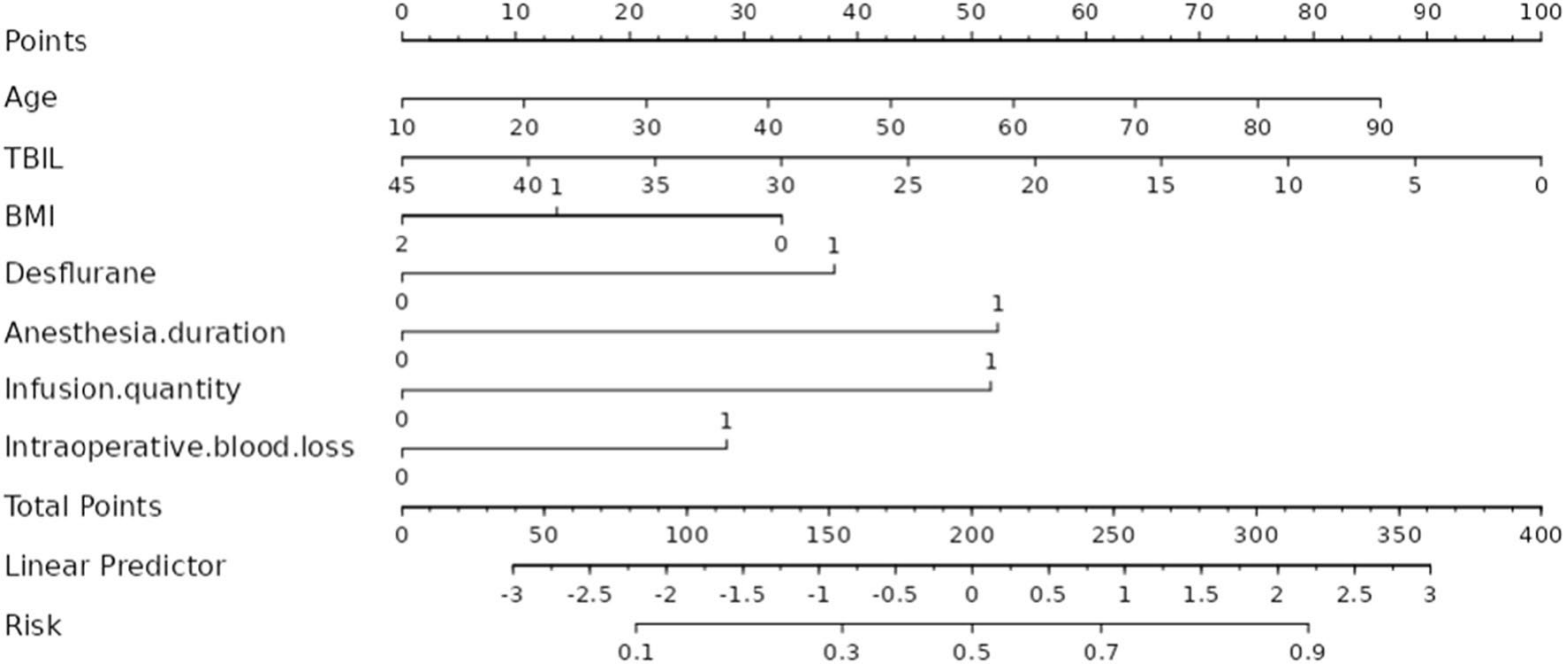
A nomogram model of intraoperative hypothermia in patients undergoing VATS lobectomy.

The model regression equation constructed according to the Logistic regression analysis results is as follows: Logit (*P*) = − 1.444 + 0.020 × Age − 0.041 × TBIL + 0.708 × Inhaled desflurane + 0.974 × Anesthesia duration + 0.962 × Intraoperative infusion volume + 0.530 × Intraoperative blood loss—0.622 × BMI (> 23.9 kg/m^2^). A nomogram model was constructed to predict the risk of intraoperative hypothermia in patients undergoing VATS lobectomy. Figure 4 has shown a nomogram model.

### Evaluation of prediction efficiency of nomogram model

**Figure 5:**
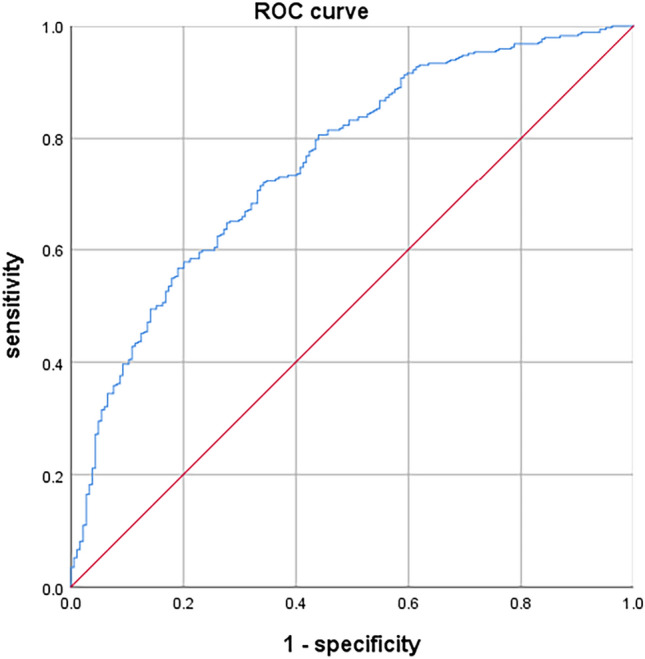
Receiver operating characteristic curve of prediction model.

**Figure 6:**
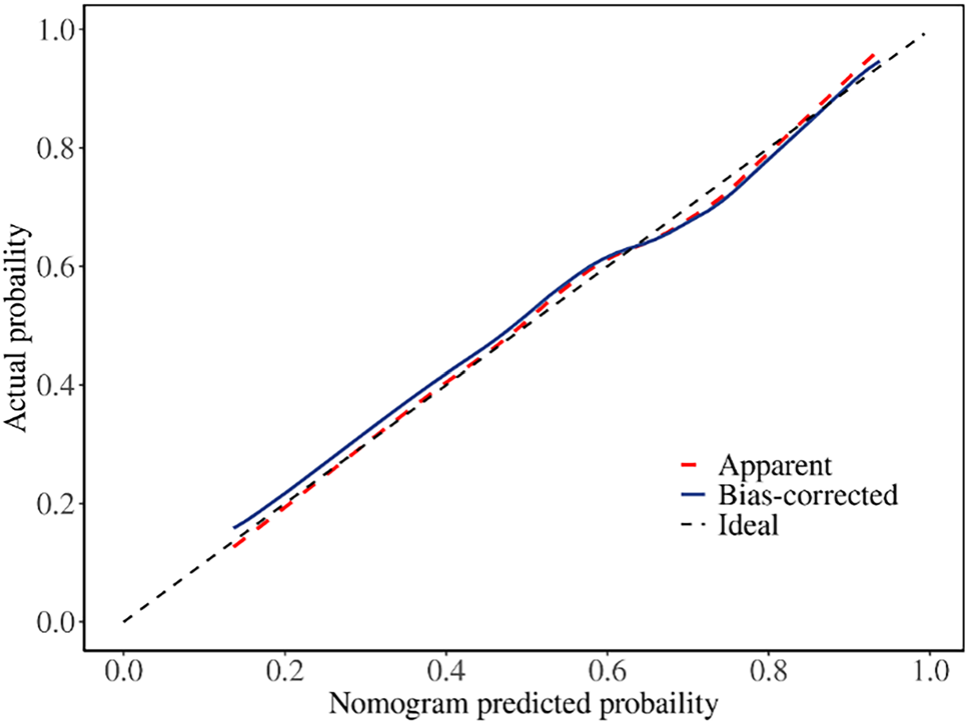
Calibration curve of prediction model.

Bootstrap method was used to repeat 1000 times for internal validation of the nomogram model. ROC curve was used to evaluate the discrimination of the model. The area under ROC curve was 0.757, 95% CI (0.714–0.799). The optimal cutoff value was 0.635, the sensitivity was 0.717, and the specificity was 0.658. These results suggested that the model was well discriminated. Calibration curve has shown that the actual values are generally in agreement with the predicted values. Hosmer–Lemeshow test showed that χ2 = 5.588, *P* = 0.693, indicating that the model has a good accuracy. The ROC curve is shown in Fig. 5. The calibration curve is shown in Fig. 6.

### Evaluation of clinical utility of nomogram model

**Figure 7:**
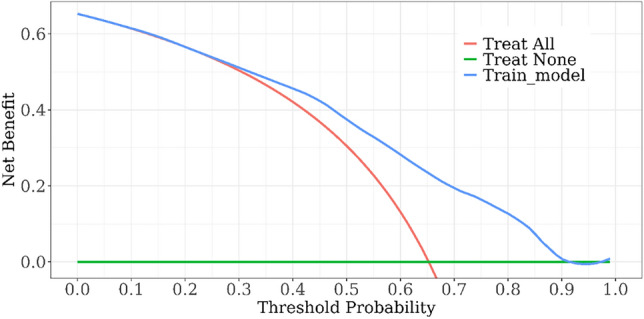
Decision curve analysis of prediction model.

DCA was used to evaluate the clinical utility of the model. DCA is to determine the clinical utility of the prediction model by calculating the net benefit of each patient developing the risk threshold of intraoperative hypothermia^27^. Figure 7 shows the DCA. The x-axis represents threshold probability, and the y-axis represents net benefit. The results have shown that the model threshold probability is between 0 and 0.90, and the net benefit rate is greater than 0, which indicates that the model has a high clinical utility.

## Discussion

This study firstly developed a model for intraoperative hypothermia prediction in patients undergoing VATS lobectomy. It can provide reference for scientific temperature management in patients undergoing VATS lobectomy.

In this study, the incidence of intraoperative hypothermia in patients undergoing VATS lobectomy was 65.28%, which was at a high level^11–13^. The reasons could be as follows: First, patients undergoing VATS lobectomy are routinely placed in a lateral position, exposing more body parts to the environment and losing more heat than supine position surgery. Second, the median age of the participants in this study was 63.00 years old, which indicates that the number of elderly patients is high. It has been reported that elderly patients are more likely to have intraoperative hypothermia than young patients^22^. Third, 80.57% (427 cases) of patients had anesthesia for more than 3 h. It has been reported that the core body temperature of the patient will gradually decrease as the time of anesthesia is prolonged^28^. In addition, anesthetics can significantly impair normal autonomic thermoregulatory control, further affecting the patient’s intraoperative body temperature^16^.

The results of this study showed that the older the age, the more likely to develop intraoperative hypothermia, which is consistent with a previous study^22^. As patients age, their basal metabolism decreases, blood circulation slows and sensitivity to temperature changes decreases^29^. In addition, elderly patients often suffer from basic diseases such as hypertension, diabetes and coronary heart disease, and their autoimmune function is poor, so they are more prone to intraoperative hypothermia. One study showed that patients with higher body fat percentage were less likely to develop intraoperative hypothermia, which is consistent with the results of this study (BMI > 23.9 kg/m^2^)^30^. Body fat acts as insulation, helping the body retain heat^13^. In overweight patients (BMI > 23.9 kg/m^2^), when the body temperature is reduced, patients with more body fat are more likely to stimulate vasoconstriction to reduce heat conduction to peripheral tissues, so as to maintain the body heat balance^31^. Therefore, these patients are not prone to intraoperative hypothermia. It is suggested that the medical staff should evaluate the BMI of the patients undergoing VATS lobectomy before surgery, and take stronger insulation measures for the patients with low body fat percentage, such as opening circulating hot water blanket and heating machine.

This study is the first to identify preoperative low serum TBIL levels and intraoperative inhaled desflurane as risk factors for intraoperative hypothermia in patients undergoing VATS lobectomy. This provides inspiration for future research on risk factors of intraoperative hypothermia. Serum TBIL, as an endogenous antioxidant in vivo, can produce anti-atherosclerosis properties through its antioxidant potential, and then provide certain protection against cardiovascular diseases^32^. The heart is the second largest energy user in the body, and half of the energy used is used to maintain body temperature. Therefore, higher serum TBIL levels can indirectly maintain body temperature. In addition, low total bilirubin is commonly seen in patients with malnutrition, anemia and other conditions. These diseases can indirectly lead to intraoperative hypothermia. Desflurane, as an inhaled anesthetic inducer, can enter the human body through the respiratory tract in the form of volatile gas, enter the blood through the alveolar artery, and with the blood circulation to the brain, block nerve transmission, and finally produce anesthesia^33^. It has been reported that desflurane inhibits the hypothalamic thermoregulatory center, reduces the temperature regulation ability of the autonomic nervous system, and slows down vasoconstriction, which increases heat loss and ultimately leads to lower body temperature in patients^34^. One study reported that desflurane inhibited vasoconstriction and chills to a greater extent when used in conventional concentrations than when propofol was administered intravenously^33^. Therefore, medical staff should pay attention to the anesthesia induction method of patients and take insulation measures in advance.

The odds ratio (OR) for the anesthesia duration was the largest among all risk factors (OR = 2.648), indicating the greatest advantage in predicting the effect of intraoperative hypothermia. When the anesthesia duration was greater than 180 min, the incidence of intraoperative hypothermia was higher in patients undergoing VATS lobectomy, which was consistent with the result of a previous study^28^. It has been reported that unheated anaesthetic can directly cause the core body temperature of patients to decrease by 1–2 °C^16^. With the extension of anesthesia duration, the redistribution rate of heat from the core to the periphery will be accelerated, resulting in a significant increase in the incidence of hypothermia. This suggests that managers should improve the operating efficiency of the operating room, optimize the operation and anesthesia process, shorten the anesthesia duration of patients, and reduce the redistribution of heat.

The results of this study showed that intraoperative infusion volume greater than 1500 ml and intraoperative blood loss greater than 100 ml were risk factors for intraoperative hypothermia in patients undergoing VATS lobectomy, which was consistent with the results of previous studies^28,35^. It has been reported that a large amount of cold liquid into the body will absorb body heat, causing patients to have chills, thereby increasing the risk of intraoperative hypothermia^36^. Excessive blood loss during the operation will not only take away a lot of heat from the body, but also increase the amount of fluid replenishment during the operation and reduce the core body temperature. However, intraoperative hypothermia may also lead to potential intraoperative bleeding^37^.

Implications for clinical practice. The nomogram model constructed in this study for the first time has good discrimination, accuracy and clinical utility in predicting patients with intraoperative hypothermia. In clinical practice, nursing staff can evaluate and quantify the risk probability of intraoperative hypothermia in patients undergoing VATS lobectomy according to this nomogram model. The risk of intraoperative hypothermia was inferred based on the patient’s age, BMI, serum TBIL levels, anesthesia duration, whether desflurane was inhaled, intraoperative infusion volume, and intraoperative blood loss, and individualized interventions were taken. For example, pay attention to the elderly and low BMI patients, and actively take measures to keep warm; the results of preoperative biochemical examination were monitored, and passive combined with active heat preservation measures were taken for patients with low serum TBIL levels^38^ (Forced draft preheating, intraoperative fluid infusion and pleural flush were heated); before anesthesia induction, the patient was pre-insulated for at least 10 min and a closed respiratory circuit system was used. One study reported that 60 min after the start of surgery is the best intervention time window to prevent hypothermia in patients undergoing VATS surgery, and appropriate insulation measures should be selected within this time window^39^. We believe that the above preventive measures can reduce the incidence of intraoperative hypothermia in patients undergoing VATS lobectomy, reduce the intraoperative, postoperative and anesthesia complications, and improve the prognosis.

This study has several limitations. First, serum TBIL levels and inhaled desflurane as risk factors for intraoperative hypothermia have not been reported, and the results need to be further verified. But this result illustrates the innovation of the study. Second, this study is a single-center retrospective study, and a multi-center longitudinal study can be conducted in the future. Third, there may be some risk factors that are not included in the model for analysis, such as psychological factors of patients.

## Conclusion

The nomogram model constructed in this study showed good discrimination, accuracy and clinical utility in predicting patients with intraoperative hypothermia, which can provide reference for medical staff to screen high-risk of intraoperative hypothermia in patients undergoing VATS lobectomy. This model is worthy of clinical promotion.

## Data Availability

The datasets used and/or analysed during the current study available from the corresponding author on reasonable request.

## Abbreviations

VATS: Video-assisted thoracoscopic
ROC: Receiver operating characteristic
DCA: Decision curve analysis
BMI: Body mass index
ASA: American society of anesthesiologists
TBIL: Total bilirubin
SD: Standard deviation
OR: Odds ratio
